# Variants in the *WDR45* Gene Within the OPA-2 Locus Associate With Isolated X-Linked Optic Atrophy

**DOI:** 10.1167/iovs.64.13.17

**Published:** 2023-10-11

**Authors:** Inbal Gazit, Idan Hecht, Chen Weiner, Alina Kotlyar, Zina Almer, Erez Bakshi, Lior Or, Hadas Volkov, Barak Feldman, Idit Maharshak, Marina Michelson, Nitza Goldenberg-Cohen, Eran Pras

**Affiliations:** 1 Department of Ophthalmology, Shamir Medical Center, Zerifin, Israel; 2 Faculty of Medicine, Tel Aviv University, Tel Aviv, Israel; 3 The Matlow's Ophthalmo-genetics Laboratory, Shamir Medical Center, Zerifin, Israel; 4 Department of Ophthalmology, Edith Wolfson Medical Center, Holon, Israel; 5 Institute of Medical Genetics, Wolfson Medical Center, Holon, Israel; 6 The Genetic Institute of Maccabi Health Medicinal Organization, Tel Aviv, Israel; 7 Rappaport Faculty of Medicine, Technion-Israel Institute of Technology, Haifa, Israel, and the Department of Ophthalmology, Bnai Zion Medical Center, Haifa, Israel

**Keywords:** optic atrophy, hereditary optic atrophy, X-linked, ADOA, LHON, WDR45

## Abstract

**Purpose:**

To describe clinical and molecular findings of two families with X-linked optic atrophy and present two new pathogenic variants in the *WDR45* gene.

**Methods:**

Case series and molecular analysis of two families of Jewish Ashkenazi descent with early onset bilateral optic atrophy. Whole-exome sequencing (WES) and bioinformatic analysis were performed, followed by Sanger sequencing and segregation analysis.

**Results:**

In both families, male siblings (three in family 1, two in family 2) had early-onset isolated bilateral optic atrophy. The sibling's healthy mother (and in the second family also one healthy sister) had a mild presentation, suggesting a carrier state and an X-linked inheritance pattern. All participants were otherwise healthy, apart from mild learning disabilities and autism spectrum disorder in two siblings of the second family. Variants in known optic atrophy genes were excluded. Analysis revealed a point variant in the *WDR45* gene—a missense variant in the first family, NM_001029896.2:c.107C>A; NP_001025067.1:p.Pro36His (variant ID: 1704205), and a splice site variant in the second family, NM_001029896.2:c.236-1G>T; NP_009006.2:p.Val80Leu (variant ID: 1704204), located on Xp11.23 (OPA2 locus). Both variants are novel and predicted as pathogenic. In both families, the variant was seen with full segregation with the disease, occurring in all affected male participants and in one allele of the carrier females, as well as none of the healthy participants.

**Conclusions:**

Among two families with isolated X-linked optic atrophy, molecular analysis revealed novel variants in the *WDR45* gene in full segregation with the disease. This gene resides within the *OPA2* locus, previously described to associate with X-linked optic atrophy. Taken together, these findings suggest that certain pathogenic variants in the *WDR45 g*ene are associated with isolated X-linked optic atrophy.

Hereditary optic atrophy is a bilateral degeneration of the optic nerves, causing insidious visual loss, typically starting at a young age. Affected individuals can have severely reduced vision, which can significantly affect their quality of life and function. The two most common inherited optic neuropathies are autosomal dominant optic atrophy (ADOA)^1^ and Leberʼs hereditary optic neuropathy (LHON),^2^ but autosomal recessive^3^^–^^5^ and X-linked cases have also been described.^6^^,^^7^

ADOA is characterized by an indolent, slowly progressive, bilateral vision loss that usually starts in the first to second decades of life.^1^^,^^2^ The most common gene associated with isolated ADOA is *OPA1*,^8^^,^^9^ followed by variants in the genes *WFS1*, *ACO2*, *SPG7*, *MFN2*, *AFG3L2*, *NR2F1*, and *FDXR*.^10^^–^^15^

Autosomal recessive optic atrophy is rare and usually a syndromic presentation that includes Wolfram syndrome (*WFS1* gene),^16^ *ACO2* gene, *RTN4IP1* (*OPA10*), *TMEM126A* gene (*OPA7*), Behr syndrome (*OPA1*), and Costeff syndrome (*OPA3*).^17^^–^^20^

LHON is usually diagnosed in the second to third decades of life and is characterized by acute or subacute episodes of unilateral vision loss, followed by vision loss in the fellow eye.^3^^,^^21^ Genetically, it is a maternally transmitted disease that results from variants in the mitochondrial genome or, in some cases, a result of autosomal recessive variants.^21^

X-linked optic atrophy (OMIM #311050) was previously described in several families in the literature.^6^^,^^7^ In two of these families, linkage studies revealed that the disease gene is localized to the *OPA2* locus on Xp11.4-Xp11.2.^6^^,^^7^ However, the causative gene has yet to be described.

The aim of this study is to characterize two families with hereditary optic atrophy with an apparent X-linked inheritance pattern clinically and molecularly. Ophthalmic and systemic findings are documented, and ophthalmic imaging together with neuroimaging are employed. Molecular analysis was used to identify the causative variants in both families.

## Materials and Methods

The study protocol was approved by the Institutional Review Board of Shamir Medical Center. Written informed consent was obtained from all participants prior to enrollment. The study protocol and consent procedures conformed with the tenets of the Declaration of Helsinki and local ethical guidelines.

**Figure 1.**
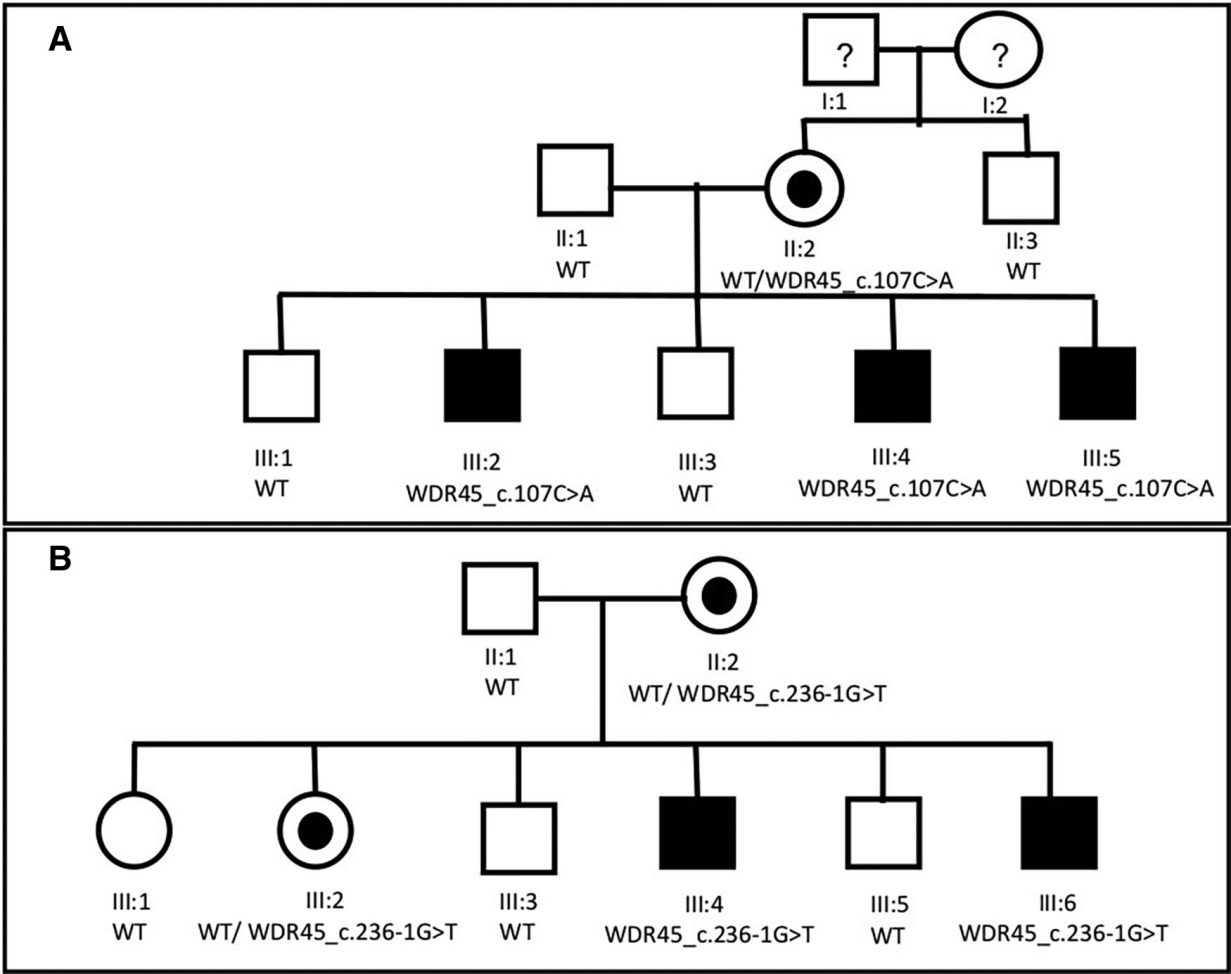
Pedigrees of the families: (**A**) family 1; (**B**) family 2. Shown are the pedigrees of both families. In family 1, participants I:1 and I:2 were not recruited or examined and are shown for clarity purposes. Clinical and molecular details are available in Tables 1 and 2.

Eight individuals from a family of Jewish Ashkenazi descent and 8 individuals from a second family, also of Jewish Ashkenazi descent, were enrolled. Pedigrees of the two families are shown in Figure 1. Recruitment took place at the Genetic Eye Clinic (Shamir Medical Center, Zerifin, Israel).

Clinical evaluation of the two families was performed at the Ophthalmology Department of Shamir Medical Center. It included a full ophthalmologic evaluation, including visual acuity, slit-lamp biomicroscopy (Haag Streit International, Koeniz, Switzerland), automated perimetry (Humphrey Field Analyzer II Model 750; Zeiss–Humphrey Systems, Dublin, CA, USA), and optical coherence tomography examination of the retinal nerve fiber layer (RNFL) and ganglion cell layer (GCL; OCT2, Heidelberg Engineering GmbH, Heidelberg, Germany, and Cirrus HD-OCT RNFL and ONH, Zeiss–Humphrey Systems). Systemic evaluations included physical examination and routine blood tests. Neuroimaging was used in selected cases to rule out intracranial or orbital findings; in particular, magnetic resonance imaging (MRI) was used to rule out iron accumulation in the brain. Demographic information and any systemic and ocular medical and surgical histories were documented.

Molecular analysis of the two families was performed at the Matlow's Ophthalmo-Genetic Laboratory, Shamir Medical Center, Israel. Genomic DNA was extracted from peripheral whole blood, using standard protocols.

Analysis included whole-exome sequencing (WES) on four family members of the first family (two affected males and their parents) with bioinformatic analysis using the Variantyx Genomic Intelligence platform (Variantyx, Framingham, MA, USA) and one family member in the second family (one affected male). WES was performed by a commercial company (Psomagen, Rockville, Maryland, USA) using the Illumina NovaSeq platform, Agilent SureSelect v6 (average coverage was about 149 reads). For direct Sanger sequencing, primers were designed using Primer 3 (https://bioinfo.ut.ee/primer3/), and sequencing was carried out at a commercial sequencing service (Macrogen Europe, Amsterdam, Netherlands) using the ABI BigDye Terminator v3.1 Cycle sequencing kit (Applied Biosystems, Waltham, Massachusetts, USA) in a Mastercycler pro 384 sequencer (Eppendorf, Hamburg, Germany). This was followed by Sanger sequencing, which was also performed on samples from the remaining family members from both families (eight family members from each family). Analysis of samples from one family member in the second family (patient III:6) was done in a private setting (MNG Laboratories, Medical Neurogenetics LLC, Atlanta, GA, USA). Sanger sequencing was used for verification of identified variants, segregation analysis within families, and exclusion of other candidate genes. The Variantyx Genomic Intelligence platform was used for bioinformatic analysis. Online bioinformatic tools (Ensembl Variant Effect Predictor, MutationTaster, Franklin by Genoox, and Varsome Human genomics) were used to examine the variants’ rarity and its effect on the protein function, as well as to evaluate evolutionary conservation.

### Variant Prioritization Strategy

Analysis of the affected patients from the first family yielded 98,084 variants in 17,215 genes with quality and depth >21. Next, we filtered by population frequency using the Exome Aggregation Consortium data (ExAc = very rare; 0%–1%), which yielded 3,768 variants in 2,486 genes. Next, we filtered by an X-linked inheritance pattern and found 357 variants in 165 genes. Excluding variants that are synonymous yielded 293 variants in 144 genes. Finally, we filtered variants by their classification prediction and excluded any that we predicted to be benign or likely benign. Variants in two genes remained, *WDR45A* and *ALG13*.

At this point, segregation analysis of the identified variants in each of the genes was performed by Sanger sequencing of members of the whole family with specifically designed primers (Primer 3). The results showed that one of the heathy brothers (III-1, family 1) had the same variant in ALG13 (p.Ser902Leu ; db781064025) as the affected brothers, and thus this gene was excluded. *WDR45A* remained the sole candidate gene.

Copy number variation (CNV) analysis was done using the CNVkit v0.7.3 with default settings.^22^ The sequencing coverage for each member of family 1 was individually calculated and normalized within the targeted exome panel regions. CNV calling was carried out using the circular binary segmentation algorithm.^39^ We subsequently annotated the copy number segments to genes and designated regions bearing a log_2_ ratio of at least ±0.4 as suggestive of shallow deletions or gains. Segments with a log_2_ ratio of less than –1.2 were classified as deep deletions, while those with a log_2_ ratio of greater than 2 were considered amplifications.^40^

## Results

Two families with hereditary optic atrophy were examined. Both are two-generation families of Ashkenazi Jewish ancestry, and eight family members were available for examination in each. Their clinical and molecular findings are detailed below separately.

### Clinical Findings

#### Family 1

**Figure 2.**
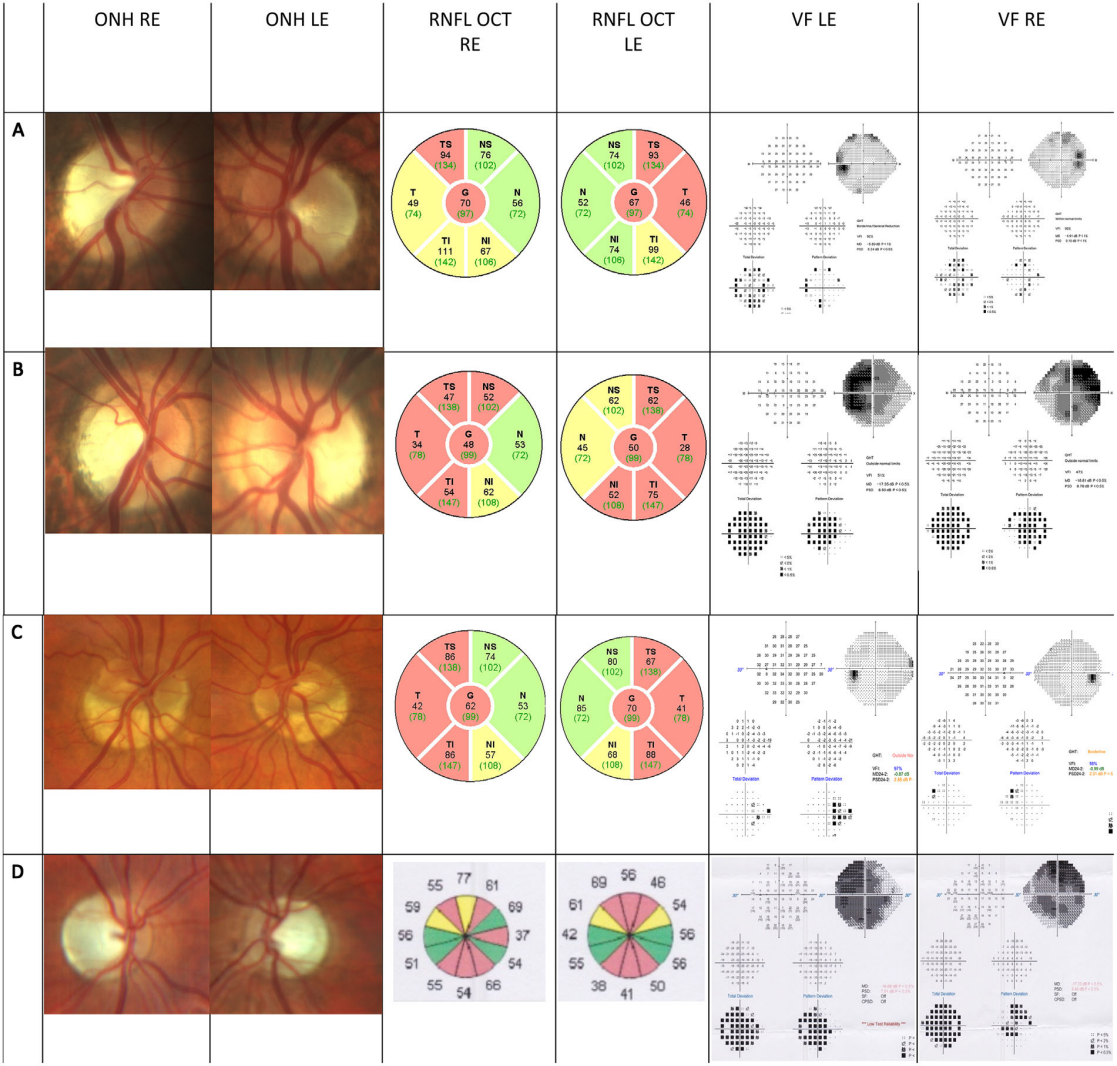
Clinical findings. Clinical findings of selected patients from families 1 and 2. (**A**, **C**) The mothers from both families (**A**, patient II:2, family 1; **C**, patient II:2, family 2). Notice the relatively preserved visual fields with thinning of RNFL thickness. (**A**) RE ONH presents with mild pallor; (**C**) tilted myopic ONH thus difficult to notice pallor. (**B**, **D**) Affected males from families 1 and 2 (**B**, patient III:3, family 1; **D**, patient III:6, family 2). Notice the visual field loss, prominent optic nerve pallor, and RNFL thinning. (**D**) Largely cupped optic disc. ONH, optic nerve head; VF, visual field.

Family 1 is of Ashkenazi Jewish ancestry. Eight family members were examined, and their clinical and molecular findings are summarized in Table 1 and the pedigree is illustrated in Figure 1A (participants I:1 and I:2 seen in the pedigree were not recruited or examined and are shown for clarity purposes). Three of five male siblings (patients III:2, III:3, and III:5) had early-onset isolated bilateral optic atrophy; their ages are 22, 18, and 7 years, respectively. All three presented with isolated bilateral decreased visual acuity (20/30–20/100 Snellen) since early childhood, which progressed gradually. Clinical examination revealed bilateral optic nerve pallor, as depicted in Figure 2. In all three participants, automated perimetry showed an extensive central scotoma and an optical coherence tomography (OCT) exam revealed decreased central RNFL and GCL thickness (see supplemental results). They were otherwise healthy with no neurologic or systemic findings, and neuroimaging (MRI) did not reveal any intracranial abnormalities and ruled out iron accumulation.

**Table 1.** Participant Characteristics and Molecular Findings of Family 1

		BCVA (Snellen)		ON Atrophy		Visual Field Defect					
Participant	Gender, Age (Y)	OD	OS	OD	OS	OD	OS	Macular Findings	VEP Results	MRI Results	Molecular Outcomes
II:1	Male, 46	20/25	20/25	–	–	–	–	–			WT
II:2	Female, 45	20/20	20/20	+	+	+	+	–	–		WDR45_c.107C>A/WT
II:3	Male, 47	20/20	20/20	–	–	–	–	–			WT
III:1	Male, 24	20/20	20/20	–	–	–	–	–			WT
III:2	Male, 22	20/50	20/63	++	++	++	++	–	++		WDR45_c.107C>A
III:3	Male, 18	20/30	20/40	++	++	++	++	–	++	–	WDR45_c.107C>A
III:4	Male, 13	20/25	20/30	–	–	–	–	–			WT
III:5	Male, 7	20/63	20/100	++	++	++	++	–	++		WDR45_c.107C>A

Clinical characteristics of the first family along with molecular findings. All affected males were hemizygous to the mutation, the mildly affected mother was heterozygous, and unaffected males carried the wild-type allele. + indicates positive finding (optic nerve atrophy or visual field defect) at a mild degree, ++ indicates a highly positive finding (clear optic nerve atrophy or severe visual field defects, reduced VEP response), and – indicates negative finding (i.e., normal optic nerve appearance, no clear visual field defects, and no macular abnormalities on OCT, normal VEP response, normal MRI). BCVA, best-corrected visual acuity; ON, optic nerve; VEP, visual evoked potentials; WT, wild type.

Patient II:2 is the siblings’ apparently healthy mother aged 45 years. She had normal visual acuity (20/20 Snellen OU). Her examination showed evidence of temporal pallor of the optic disc bilaterally and mild decrease in temporal RNFL thickness. Automated perimetry showed diffuse decrease in sensitivity with no specific area of scotoma. These findings are seen in Figure 2. No other systemic or neurologic findings were seen. Her mild presentation raised the possibility of a X-linked inheritance pattern. The father (II:1), the two remaining healthy siblings (III:1, III:4), and the mother's healthy brother (II:3) had a normal ophthalmic examination, intact visual fields, and normal RNFL thickness.

#### Family 2

Family 2 is also of Ashkenazi Jewish ancestry. Eight family members were examined, and their clinical and molecular findings are summarized in Table 2 and the pedigree is illustrated in Figure 1B. Two male siblings (patients III:4, III:6) out of six siblings (an additional two females and two males) were examined due to early-onset isolated bilateral optic atrophy. Their ages are 32 and 26 years; both presented with isolated bilateral decreased visual acuity (20/400–20/100 Snellen) since early childhood, which progressed gradually. Clinical examination revealed bilateral optic nerve pallor. In patient III:6, largely capped optic nerve head was noticed but glaucoma was ruled out. In both participants, automated perimetry showed extensive central scotoma and an OCT exam revealed decreased central RNFL thickness. Visual evoked potential exams showed low amplitudes with increased latency in both (see supplemental results). Patient III:4 has been diagnosed with autism spectrum disorder and bipolar disorder. Patient III:6 has learning disabilities (dyscalculia and dyslexia) as well as attention-deficit/hyperactivity disorder. Both had no other neurologic or systemic findings, and neuroimaging (MRI) did not reveal any intracranial abnormalities or iron accumulation.

**Table 2.** Participant Characteristics and Molecular Findings of Family 2

		BCVA (LogMAR)		ON Atrophy		Visual Field Defect					
Participant	Gender, Age (Y)	OD	OS	OD	OS	OD	OS	Macular Findings	VEP Results	MRI Results	Molecular Outcome
II:1	Male, 65	20/25	20/25	–	–	–	–	–			WT
II:2	Female, 60	20/25	20/30	+	+	+	+	–	–		WDR45_c.238G>T/WT
III:1	Female, 44	20/20	20/20	–	–	–	–	–			WT
III:2	Female, 42	20/30	20/25	+	+	+	+	–	–		WDR45_c.238G>T/WT
III:3	Male, 40	20/20	20/20	–	–	–	–	–			WT
III:4	Male, 32	20/150	20/150	++	++	++	++	–	++	–	WDR45_c.238G>T
III:5	Male, 30	20/20	20/20	–	–	–	–	–			WT
III:6	Male, 26	20/150	20/150	++	++	++	++	–	++	–	WDR45_c.238G>T

Clinical characteristics of the second family along with molecular findings. All affected males were hemizygous to the mutation, the mildly affected mother was heterozygous, and unaffected males carried the wild type allele. + indicates positive finding (optic nerve atrophy or visual field defect) at a mild degree, ++ indicates a highly positive finding (clear optic nerve atrophy or severe visual field defects, reduced VEP response), and – indicates negative finding (i.e., normal optic nerve appearance, no clear visual field defects, and no macular abnormalities on OCT, normal VEP response, normal MRI).

Patient II:2 from family 2 is the siblings’ apparently healthy mother aged 60 years. She had preserved visual acuity, mild myopia, and esotropia treated with prisms. Her examination showed mild disc pallor. No other systemic or neurologic findings were seen. One healthy sister, aged 42 years, also had a mild presentation similar to the mother, with preserved visual acuity and disc pallor on examination. The father (II:1), the two remaining healthy brothers (III:5, III:6), and the other healthy sister (III:1) had a normal ophthalmic examination and intact visual acuity. Intraocular pressure was measured in all patients of both families and glaucoma was ruled out as the cause of optic atrophy. None of the affected patients reported having any kind of seizures during their lifetime.

### Molecular Findings

**Figure 3.**
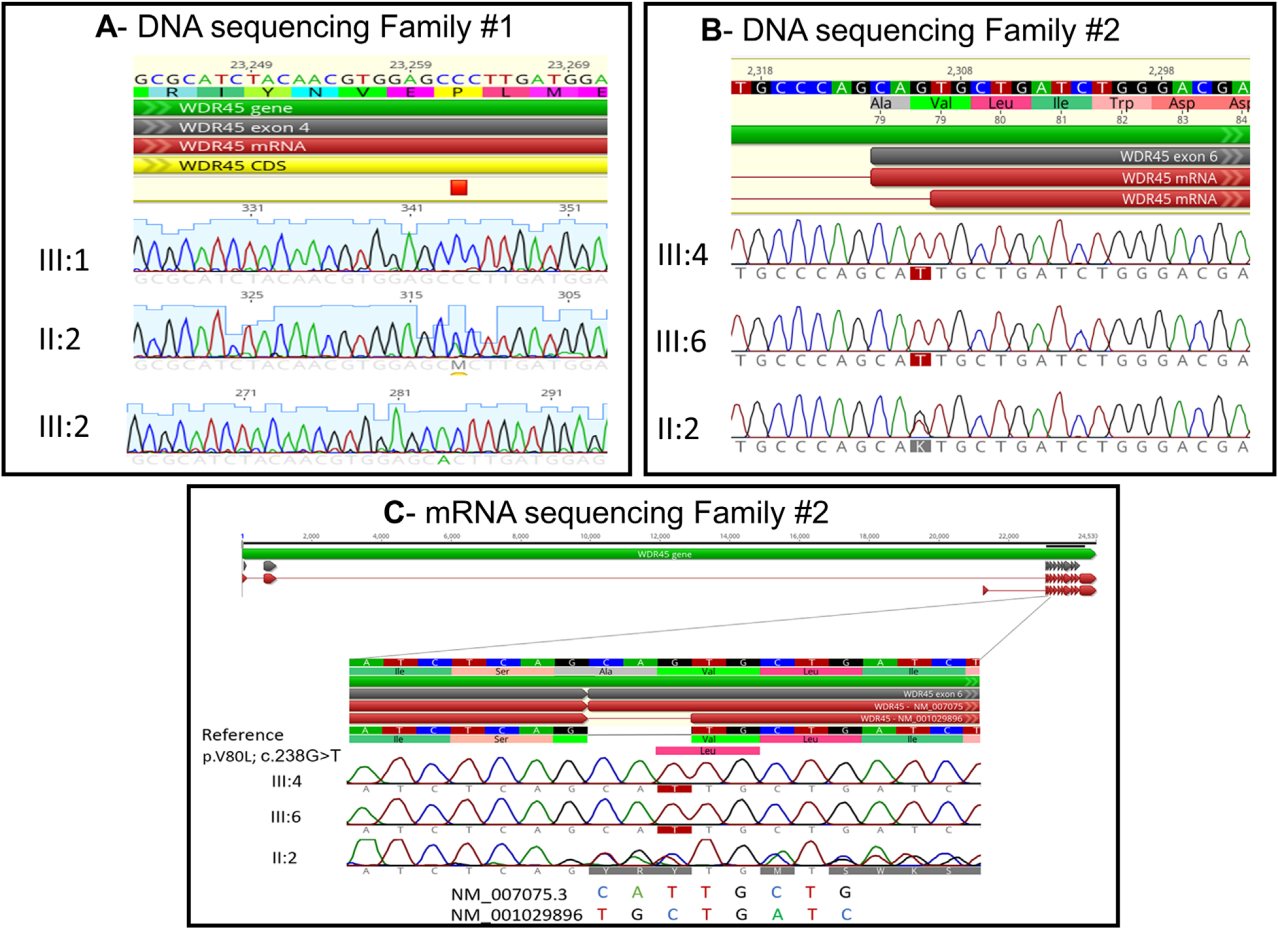
Molecular findings. (**A**) Genomic sequence of the first family shows the position of the variant. (**B**) Genomic sequence of the second family; the position of the variant is shown. Hemizygous in the affected males (III:4, III:6, and II:2 [not shown]) and heterozygous in the mother with two different transcripts from two alleles (II:2). (**C**) Messenger RNA sequencing is shown. The affected male participants express only one of the two transcripts of *WDR45*, while the mother expresses both transcripts.

First, known pathogenic gene variants were ruled out, including *OPA1*, *WFS1*, *ACO2*, *OPA3*, *OPA8*, *OPA13*, *MFN2*, and *OPA10* (Supplementary Table S1). In the first family, a missense variant in the *WDR45* gene (NM_001029896.2:c.107C>A; NP_001025067.1:p.Pro36His; variant ID: 1704205) was found in full segregation with the disease: hemizygous in the affected males and heterozygous in the mildly affected mother, while unaffected males carried the wild-type allele (Fig. 3, Supplementary Table S2). This variant is a missense variant occurring in a highly evolutionarily preserved site and is predicted to affect the protein functionality by bioinformatic predictive tools. This variant is novel and the *WDR45* gene has not previously been described to be associated with isolated optic atrophy. Importantly, the gene is located on Xp11.23, which is within the area of the *OPA2 l*ocus, previously described to be associated with X-linked optic atrophy. CNV analysis based on the WES data did not reveal a shared copy number across patient samples or any that could be confidently associated with the pathogenic features of our investigation (Supplementary Fig. S1).

Analysis of participants from the second family revealed a second variant in the *WDR45* gene, also in full segregation. The variant exists in both affected male participants and in one allele of the healthy mother and healthy sister (carriers). All healthy family members did not carry the variants, confirming full segregation.

The *WDR45* gene undergoes alternative splicing, producing multiple transcripts that encode different protein isoforms. Specifically, the transcript NM_007075 gives rise to the protein isoform NP_009006.2, while the transcript NM_001029896.2 leads to the protein isoform NP_001025067.1. As shown in Figures 3B and 3C, the affected patient carries genomic variants that impact both protein isoforms. In the protein isoform encoded by NM_007075, the variant causes the amino acid change p.Val80Leu (NP_009006.2:p.Val80Leu; variant ID: 1704204). In the protein isoform encoded by NM_001029896.2, the same nucleotide change interferes with the splice site, presumably eliminating it (NM_001029896.2:c.236-1G>T). For the sake of clarity, this is referred to as “c.238G>T,” even though the actual splice site change occurs at c.236-1G>T. The protein isoform encoded by NM_001029896.2 also carries another variant, leading to the amino acid change p.Pro36His (NP_001025067.1:p.Pro36His). Further messenger RNA sequencing (Fig. 3) showed that the patients III:4 and III:6 have only one allele, which is the long transcript with missense mutation P.V80L, while the mother II:2 has two transcripts, one long transcript with the missense mutation and a second short transcript that is the wild-type transcript. The protein model is seen in Supplementary Figure S2. According to prediction tools (I-TASSER; Supplementary Fig. S2), the variant is predicted to affect the tertiary protein structure. This variant as well has not been described in the past, and the involvement of the *WDR45* gene within the *OPA2 l*ocus is consistent with the findings in family 1.

## Discussion

In this study, we examined two families who presented with early-onset isolated bilateral optic atrophy. In the first family, the apparently healthy mother of the affected siblings presented with mild clinical findings despite no decrease in visual acuity. This raised the possibility of an X-linked inheritance pattern. This is of special interest as the exact molecular basis of isolated X-linked optic atrophy has not yet been described. Molecular analysis of both families revealed full segregation with novel variants in the *WDR45* gene. It is interesting to note that in the second family, the variant is a splice site variant affecting one of two main transcripts of *WDR45*. This finding might be clinically important, as splice site variants are potential candidates for future gene therapy by small interfering RNA.^23^^,^^24^ The *WDR45* gene has not previously been described to be associated with isolated optic atrophy, but the gene is located (Xp11.23) within the area of the *OPA2* locus.^25^ Linkage studies have previously identified this locus as responsible for the disease among families with X-linked optic atrophy, but the causative gene has yet to be identified.^6^^,^^7^ Given the results seen here, we suggest that variants in the *WDR45* gene are associated with X-linked optic atrophy.

*WDR45* variants were described in the past as X-linked dominant inherited, affecting loss of function of autophagy processes. They are associated with severe neurologic conditions such as beta-propeller protein-associated neurodegeneration (BPAN), static encephalopathy of childhood with neurodegeneration in adulthood (SENDA), Rett-like features, and infantile spasm (West syndrome).^25^^–^^30^ These severe neurologic syndromes typically present with convulsions in early childhood and adolescence, followed by young adulthood neurodegenerative regression, including cognitive regression and parkinsonism. Typical imaging finding in these syndromes is iron accumulation in the basal ganglia and substantia nigra, as an explanation to the clinical parkinsonism.^29^ Interestingly, in BPAN syndrome, ocular manifestations have been described in several patients. They include optic atrophy as well as retinal colobomas, nystagmus, high myopia, astigmatism, spontaneous retinal detachment, retinal pigmentary changes, abnormal electroretinogram with retinitis pigmentosa–like fundus, and cortical blindness.^25^^–^^28^ The variants that have been described in the aforementioned syndromes are mostly truncating variants causing lower levels of *WDR45* protein and resulting in impaired autophagy flux.^25^^–^^30^ To the best of our knowledge, the variants in *WDR45* gene found in our families have not been described in the past in any of the neurologic syndromes related to *WDR45* in the literature, confirming that these variants are novel and not partial penetrance expression of known variants. Moreover, the variants we found result in a missense change in the predicted protein rather than its truncation, which might explain the relatively mild phenotype.

The clinical findings of the patients described in our study are markedly different compared to these syndromes. None of the severe and early neurologic manifestations were seen. Most affected participants had no neurologic manifestations at all. One sibling from the second family had learning disabilities, and another had autism spectrum disorder and bipolar disorder. Whether these are a direct result of the disease, coincidental, or related to severe visual impairment since an early age is difficult to say. Neither have been previously described in association with *WDR45* dysfunction. It is also worth considering that most of our patients are young, and gait ataxia or other parkinsonian symptoms might develop in later years, and it would be interesting to follow whether they would develop such complications in the future. Regardless, compared to the severe and debilitating manifestations seen with other syndromes associated with *WDR45* variants, the phenotype seen here is essentially isolated X-linked optic atrophy.

We assume that the variants we found in the *WDR45* gene might impair autophagy processes. Autophagy is a core molecular pathway of human cells and is responsible for cell homeostasis and longevity.^31^ The mainstay of the autophagy pathway is to deliver cytoplasmic content to the lysosome for degradation. It accounts for degradation of damaged organelles, digestion of misfolded proteins, and adaption to stress conditions.^31^ Autophagy loss of function is related to many major pathologies such as the development of tumors; neurodegenerative diseases; autoimmune diseases; cardiovascular, renal, and hepatic diseases; and ocular disorders.^32^ Mouse knockout studies have shown that impaired autophagy results in intracytoplasmic aggregation and protein accumulation, leading to axonal damage in multiple neurodegenerative diseases such as Alzheimer, Parkinson, and Huntington diseases; amyotrophic lateral sclerosis; and lysosomal diseases such as Niemann-Pick and mucopolysaccharidosis.^33^ Optic atrophy has also been linked in the past to impaired autophagy via axonal degeneration of the optic nerve in various conditions such as hereditary optic atrophy, glaucoma, and traumatic optic atrophy.^33^ In ADOA, linked to the *OPA1* gene, abnormal autophagy was shown to be a main component in the pathogenesis of the disease.^34^ It is also known that *WDR45* variants in BPAN and SENDA syndromes cause lower autophagy activity and accumulation of aberrant autophagic structures.^25^^–^^30^ Here the results suggest that variants in the *WDR45* gene are linked with X-linked isolated optic atrophy. The fact that this gene is responsible for autophagy processes is consistent with other types of hereditary optic atrophy as the pathologic mechanism.

A wide spectrum of phenotypic expressions resulting from different variants of the same gene is a known phenomenon. One example for this are variants in the *NLRP3* gene on chromosome 1q44 that codes for cryopyrin.^35^^,^^36^ Different variants in this gene can result in a wide variety of syndromes ranging from mild (familial cold autoinflammatory syndrome)^36^ to intermediate (Muckle-Wells syndrome)^37^ to severe (neonatal onset multisystem inflammatory disease).^38^ In this study, we describe a new phenotype of *WDR45* gene—isolated X-linked optic atrophy, which is clinically separate from other known phenotypes, including the aforementioned severe neurologic syndromes and revealing the wide spectrum of phenotypes caused by variants in the *WDR45* gene.

Given the many known associations of optic atrophy with potentially severe neurologic manifestations in syndromes associated with *WDR45* and other genes, it is prudent to always refer children to a neurologist for complete evaluation. A major clinical challenge is the diagnosis of female carriers, which can be easily missed. Their ocular manifestations are mild, and they are mostly asymptomatic, hence challenging to identify. This is a known phenomenon of X-linked diseases, but in this context, accurately identifying carriers is crucial to correctly classify the inheritance pattern.

The limitations of this study include the small number of examined patients, from only two families, owing to the exceedingly rare nature of X-linked optic atrophy. Another limitation of the study is the fact we performed WES rather than whole genome sequencing (WGS), which does not cover deep intronic variants. Some of the carrier females had mild findings that could be considered subjective, especially as some myopic changes can also be considered for the clinical appearance. The diagnosis was made by our team of experienced ophthalmo-geneticists based on the appearance of the optic discs, which clinically showed mild optic disc pallor, together with the RNFL and GCL changes. Another limitation was that mitochondrial DNA analysis was not performed as mitochondrial DNA was not covered in our WES analysis, and so mitochondrial diseases such as LHON cannot be molecularly ruled out. However, the gradually progressing bilateral clinical course, with onset at an early age and optic atrophy, is not suggestive of LHON. Together with the inheritance pattern, which is not suggestive of mitochondrial disease, and the positive findings concerning *WDR45*, the possibility of a mitochondrial disease is less likely. Further studies should include mitochondrial DNA analysis in addition to functional studies to verify these results.

To conclude, among two families with early-onset isolated bilateral optic atrophy, an X-linked inheritance pattern was seen. Molecular analysis revealed two different pathogenic variants in the *WDR45* gene, a gene not previously described to be associated with isolated optic atrophy. This gene resides within the *OPA2* locus, previously described to be associated with isolated X-linked optic atrophy. Taken together, these findings suggest that variants in the *WDR45* gene are responsible for isolated X-linked optic atrophy.

## Supplementary Material

Supplement 1

Supplement 2

Supplement 3

Supplement 4

Supplement 5
