# Supplementary material for: Variants in the WDR45 Gene Within the OPA-2 Locus Associate With Isolated X-Linked Optic Atrophy
Source: Invest Ophthalmol Vis Sci. 2023 Oct 11;64(13):17. doi: 10.1167/iovs.64.13.17 (PMC10573587; doi:10.1167/iovs.64.13.17)
Supplement: Supplement 1 [file iovs-64-13-17_s001.pdf]

Methods used for Copy number variations analysis

Copy number segments were annotated to genes, and regions bearing a log2 ratio of at least ±0.4 were identified as suggestive of shallow deletions or gains. Segments with log2 <−1.2 were classified as deep deletions, and those with log2 >2 were classified as amplifications.

To estimate copy number variations, we utilized CNVkit v0.7.3 with default settings[21]. CNVkit determines the mean read depths for both on-target and off-target reads and then combines these for each interval. These combined mean read depths are adjusted for systematic biases, such as GC content and mappability, to derive the log2 copy number ratios.

The sequencing coverage for each member of family 1 was individually calculated and normalized within the targeted exome panel regions. Notably, we did not compare the samples to a pooled reference panel to avoid obscuring shared and inherited variants unique to the family. CNV calling was carried out using the Circular Binary Segmentation algorithm[1]. We regarded very small copy changes (≤3 bins) as likely artifacts.

Subsequently, we annotated the copy number segments to genes, and designated regions bearing a log2 ratio of at least ±0.4 as suggestive of shallow deletions or gains. Segments with a log2 ratio of less than -1.2 were classified as deep deletions, while those with a log2 ratio of greater than 2 were considered as amplifications.

Supplemental Figure 1*. CNV analysis based on the WES data*

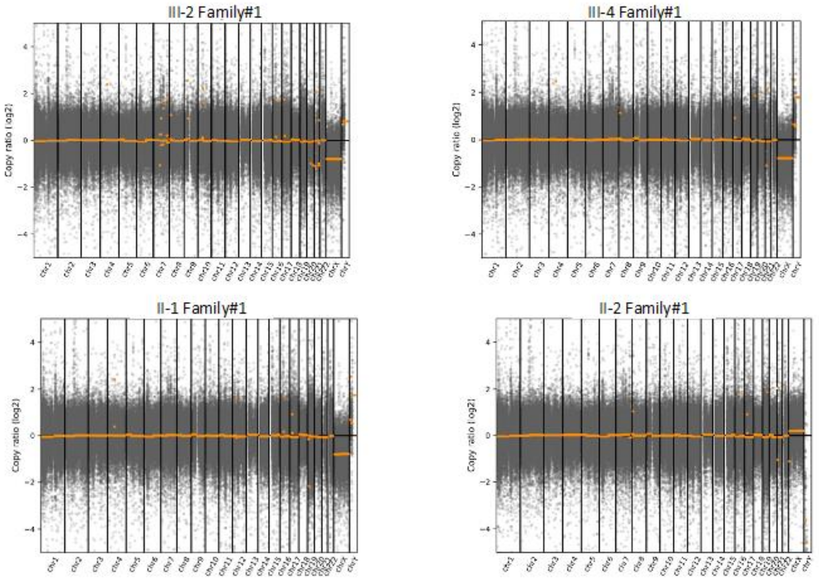

Figure Legend*: Results of copy number variation analysis based on the WES data. CNVkit v0.7.3 was used. Each sample was analyzed separately against a flat reference. As expected, gender chromosomes are heterozygous according to sample sex. No identification of cooccurrence of CNV in patient samples was detected. CNV, copy number variations; WES, whole exome sequencing.*

References:

1. Olshen AB, Venkatraman ES, Lucito R, Wigler M. Circular binary segmentation for the analysis of array-based DNA copy number data. Biostatistics. 2004 Oct;5(4):557–72. pmid:15475419
