# Supplementary material for: Variants in the WDR45 Gene Within the OPA-2 Locus Associate With Isolated X-Linked Optic Atrophy
Source: Invest Ophthalmol Vis Sci. 2023 Oct 11;64(13):17. doi: 10.1167/iovs.64.13.17 (PMC10573587; doi:10.1167/iovs.64.13.17)
Supplement: Supplement 2 [file iovs-64-13-17_s002.pdf]

Supplemental Figure 2. Changes in tertiary and quaternary protein structures caused by the *WDR45* gene mutation.

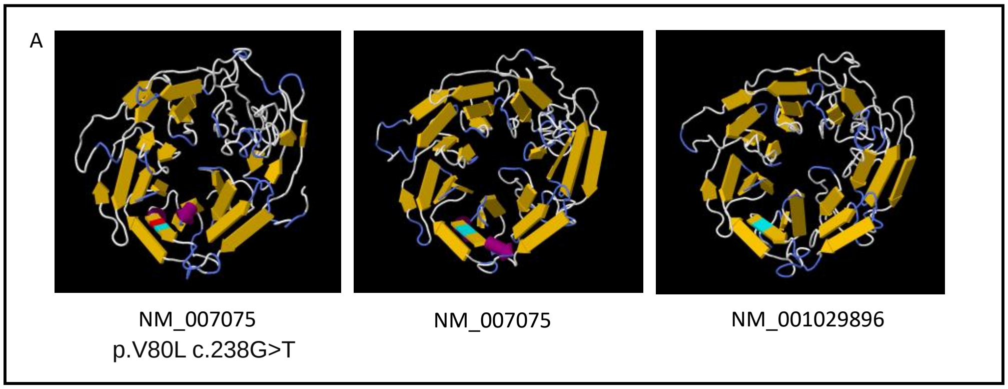

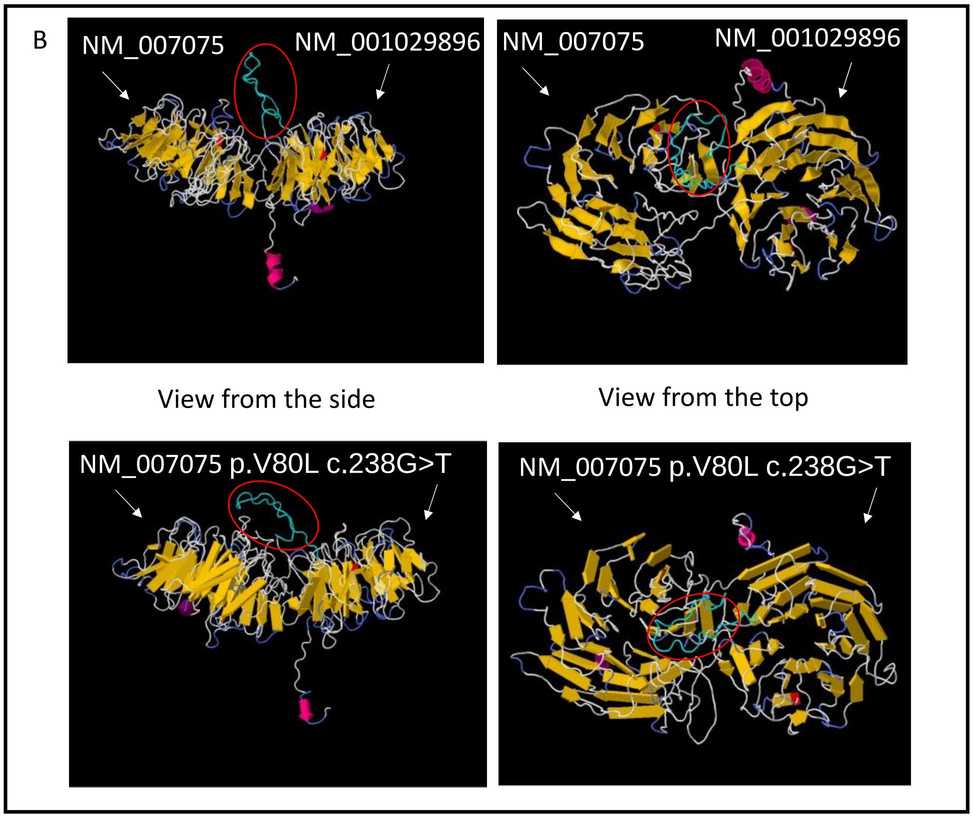

A- In red is the missense mutation p.V80L c.238G>T, Cyan is the wild-type amino acid following Leu amino acid downstream. According to I-TASSER protein structure prediction tool, the missense mutation is between two beta sheet’s structure and changes the secondary structure between the two beta sheets.

B- The red markers are Valine aa at location 80. There is an extra turn element in the tertiary structure of the mutated protein marked in cyan color and red circle.
