# Supplementary material for: Variants in the WDR45 Gene Within the OPA-2 Locus Associate With Isolated X-Linked Optic Atrophy
Source: Invest Ophthalmol Vis Sci. 2023 Oct 11;64(13):17. doi: 10.1167/iovs.64.13.17 (PMC10573587; doi:10.1167/iovs.64.13.17)
Supplement: Supplement 3 [file iovs-64-13-17_s003.pdf]

***Supplemental Table 1**. Genes known to be associated with optic atrophy and their respective coverage depth.*

Gene	*Average Coverage Depth*
OPA1	*90x*
MFN2	*159x*
SPG7	*135x*
AFG3L2	*97x*
DNM1L	*105x*
OPA3	*51x*
OPA10	*87x*
HMSN6A (SLC25A46)	*75x*
OPA13 (SSBP1)	*73x*
WFS1	*139x*
RTN4IP1	*87x*
TMEM126A	*71x*
ACO2	*135x*
