# Supplementary material for: Variants in the WDR45 Gene Within the OPA-2 Locus Associate With Isolated X-Linked Optic Atrophy
Source: Invest Ophthalmol Vis Sci. 2023 Oct 11;64(13):17. doi: 10.1167/iovs.64.13.17 (PMC10573587; doi:10.1167/iovs.64.13.17)
Supplement: Supplement 4 [file iovs-64-13-17_s004.pdf]

***Supplemental Table 2**. ACMG classification of variants*

WDR45:c.107C>A p.Pro36His<br><br>*NM_001029896*	• *PM2: Pathogenic Moderate - Extremely low frequency in gnomAD, 1000Genome or ExAc population databases.*<br>• *PP3: Moderate - Multiple lines of computational evidence support a deleterious effect on the gene or gene product (conservation, evolutionary, splicing impact, etc.) (Pathogenic, Supporting).*<br>• *PP5: Supporting - ClinVar classifies this variant as Likely Pathogenic.*
WDR45:c.236-1G>T<br><br>*NM_001029896*	• *PVS1: Pathogenic, Very Strong - splice sites*<br>• *PM2: Supporting - Extremely low frequency in gnomAD, 1000Genome or ExAc population databases.*<br>• *PP5: Supporting - ClinVar classifies this variant as Likely Pathogenic.*

Variants and their respective Interpretation according to the Joint Consensus Recommendation of the American College of Medical Genetics and Genomics and the Association for Molecular Pathology.[38] PVS criteria indicate Very strong evidence of pathogenicity. PS, Strong evidence of pathogenicity. PM, Moderate evidence of pathogenicity, and PP, Supporting evidence of pathogenicity. See Richards S et al. for details on classification groups.[38]
