# Supplementary material for: Variants in the WDR45 Gene Within the OPA-2 Locus Associate With Isolated X-Linked Optic Atrophy
Source: Invest Ophthalmol Vis Sci. 2023 Oct 11;64(13):17. doi: 10.1167/iovs.64.13.17 (PMC10573587; doi:10.1167/iovs.64.13.17)
Supplement: Supplement 5 [file iovs-64-13-17_s005.pdf]

Supplemental VEP and OCT results:

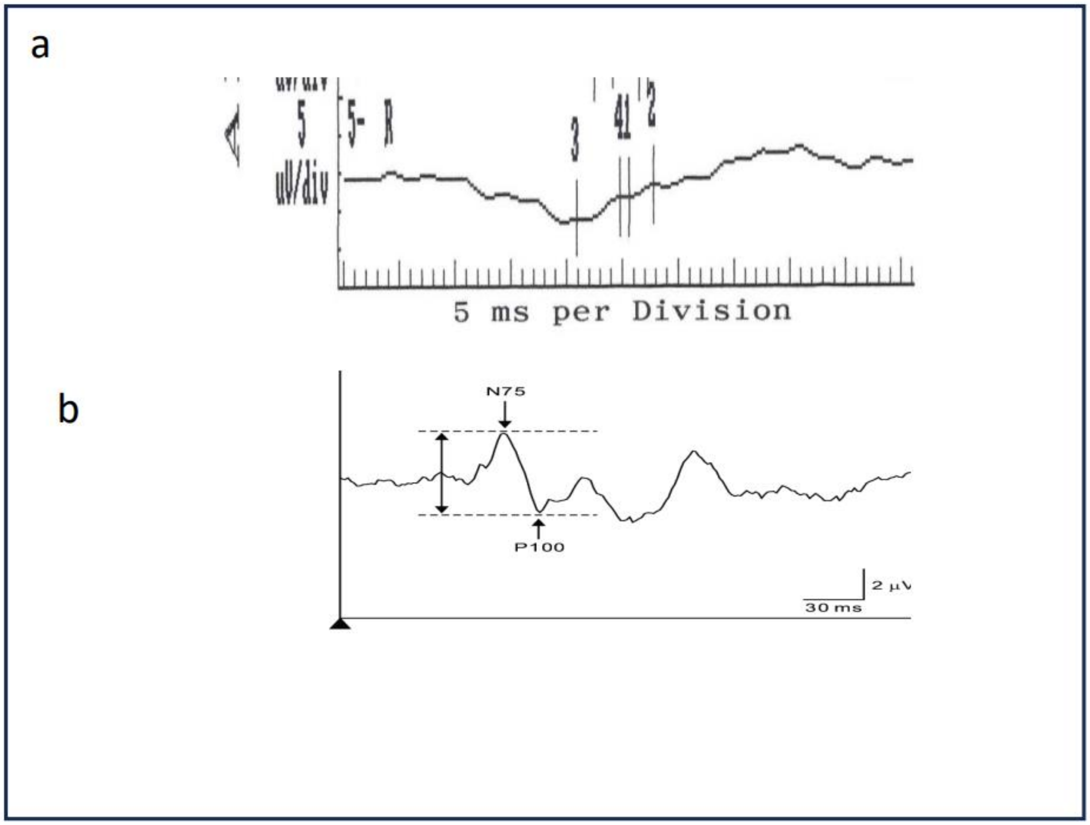

***Figure Legend**:* VEP result of patient III-6, family #2. A- VEP (patient III-6, family #2) showing latencies prolonged to 135.5 ms and amplitudes are vestigial 3.4 µV. B- Normal VEP response, minimal amplitude 7 µV, maximal latency 120 ms.

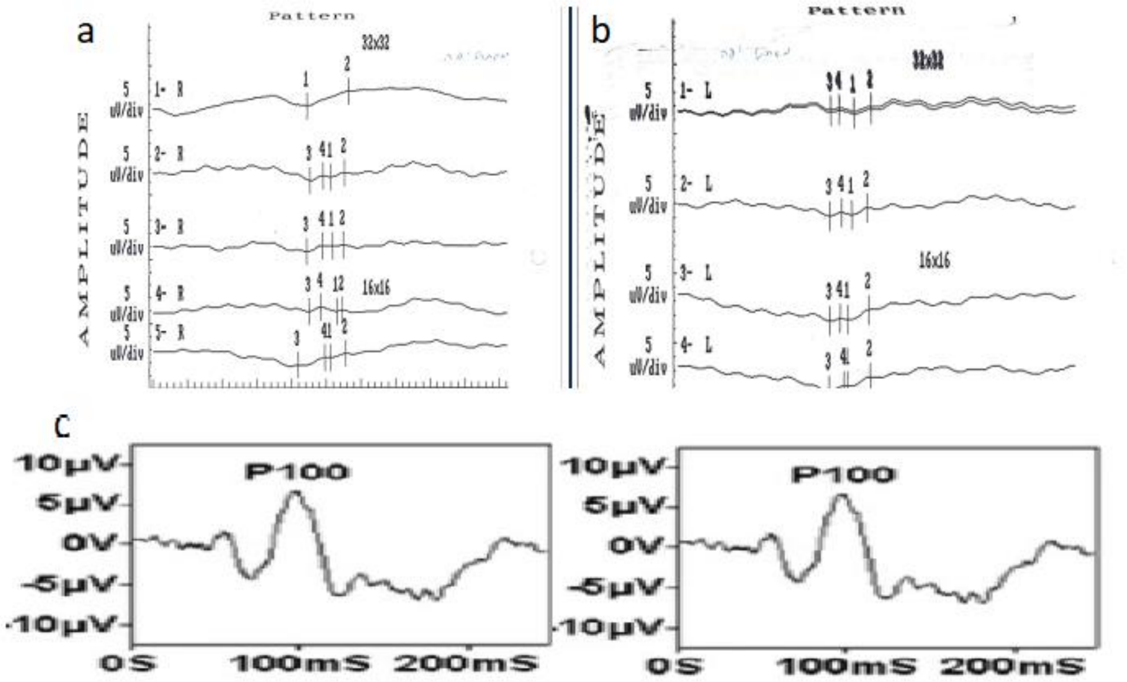

***Figure Legend**:* VEP results of patient III-6, family #2. a- Pattern VEP of the right eye; b- Pattern VEP of the left eye. Both show a flat response with no discernible positive wave. c- example of a normal pattern VEP. VEP, Visual Evoked Potential.

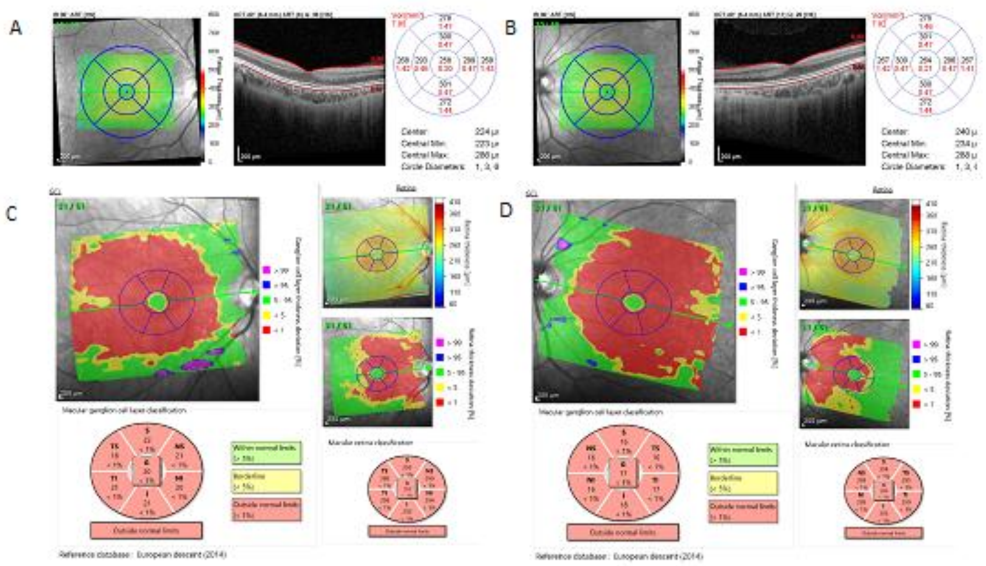

***Figure Legend**: Macular OCT and Ganglion cell complex of patient III-2 family #1. A and B- Normal macular OCT of the right and the left eyes; C and D – Ganglion cell complex of the right and the left eye showing global thinning.*
